# Crosstalk Between Histone and m^6^A Modifications and Emerging Roles of m^6^A RNA Methylation

**DOI:** 10.3389/fgene.2022.908289

**Published:** 2022-06-15

**Authors:** Zibin Xu, Tingfei Xie, Xiaolu Sui, Yunpeng Xu, Lecai Ji, Yanzi Zhang, Aisha Zhang, Jihong Chen

**Affiliations:** ^1^ Department of Nephrology, Affiliated Bao’an Hospital of Shenzhen, The Second School of Clinical Medicine, Southern Medical University, Shenzhen, China; ^2^ Department of Nephrology, The Second Affiliated Hospital of Shenzhen University, Shenzhen, China

**Keywords:** N6-methyladenosine (m^6^A), histone modification, RNA modification, RNA methylation, epigenetics

## Abstract

RNA, like DNA and proteins, has been discovered to undergo dynamic and reversible chemical alterations, increasing the diversity and functional complexity of the molecule. N-6-methyladenosine (m^6^A) RNA methylation serves as a bridge between transcription and translation and is critical for many diseases’ progression. There is a complex interrelationship between m^6^A modifications and other epigenetic modifications. Their crosstalk significantly affects transcriptional outputs, translation, recruitment of chromatin modifiers, as well as the deployment of the m^6^A methyltransferase complex at target sites. This article outlines the potential function of m^6^A RNA methylation in epigenetics and summarizes its interactions with histone modifications.

## 1 Introduction

N-6-methyladenosine (m^6^A) RNA methylation is a widespread reversible modification that occurs on RNA at the nitrogen atom at position six of the adenosine base and is conserved in several species from yeast to human, with a common base motif of DRACH (D = G/A/U; R = G/A; H = A/C/U). Although this sequence is commonly found in the transcriptome, in mammals, approximately 0.2%–0.6% of adenosine in mRNA is modified by m^6^A, with an average of 3–5 m^6^A-modified sites per transcript (Fu et al., 2014). The m^6^A RNA methylation on the DRACH motif is not randomly distributed in the transcript but is enriched in the 3′-untranslated region (UTR) near the mRNA stop codon and the exon (Shi et al., 2020). Comprehensive analysis of m^6^A and m^6^Am RNA methylation in human and mouse tissues and human cell lines revealed that m^6^A is widely present in various human and mouse tissues and has similar distribution patterns and shared motifs in all human tissues and cell lines. Brain tissue had the highest tissue specificity for m^6^A RNA methylation, and tissue-specific m^6^A modifications distinguished between different types of human and mouse tissues (Liu et al., 2020).

## 2 m^6^A Writer, Eraser, Reader

### 2.1 m^6^A Writer

m^6^A has a large methyltransferase complex (MTC), of which methyltransferase-like 3 (METTL3) is the core component, catalytically active and bound to the methyl donor S-adenosyl-L-methionine (SAM) to catalyze methyl transfer to the modification site. METTL14 acts as a metathesis agent, forming a stable heterodimer with METTL3 and co-locating in the nuclear speckles in a 1:1 ratio (Liu et al., 2014; Wang et al., 2016). Wilms tumor 1-associating protein (WTAP) facilitates the transfer of the METTL3-METTL14 complex to the m^6^A methylation site, which is also essential for nuclear speck localization (Ping et al., 2014). In addition to catalyzing m^6^A methylation modifications, cytoplasmically localized MTCs also serve as protein scaffolds in RNA processing and metabolism, e. g., METTL3 acts as a potential m^6^A reader to recognize the 3′UTR m^6^A site on mRNA and interacts with eukaryotic translation initiation factor 3H (eIF3h) to promote translation loop formation and thus transcript protein translation (Lin et al., 2016). METTL3 is also recruited to chromatin transcription-dependent and co-transcribed with nascent transcript methylation (Barbieri et al., 2017; Knuckles et al., 2017). METTL16 and METTL5 have recently been identified as new methyltransferases, which are methyltransferases that catalyze U6 spliceosomal small nuclear RNA and ribosomal RNA (Pendleton et al., 2017; Sepich-Poore et al., 2022).

### 2.2 m^6^A Eraser

The discovery of m^6^A RNA demethylases, including fat mass and obesity-associated protein (FTO) and human AlkB homolog H5 (ALKBH5), validates that m^6^A modification is dynamic and reversible. FTO is the first RNA demethylase discovered to sequentially oxidize m6A to N6-hydroxymethyladenosine and N6-formyladenosine, which are further hydrolyzed to adenine. FTO can act on a variety of substrates, and FTO demethylates m^6^A and m^6^Am on mRNA and m^1^A on tRNA (Wei et al., 2018). The demethylation activity of FTO on m^6^A is more pronounced in the nucleus than in plasma, while its demethylation activity on m^6^Am is more pronounced in the cytoplasm. Since the abundance of m^6^A in mRNA is much higher than that of m^6^Am, FTO mainly mediates the demethylation of m^6^A in cells, despite the fact that m^6^Am is the preferentially bound substrate *in vitro*. Two studies reported that the effect of cap m^6^Am on translation under basal conditions could be negligible (Boulias et al., 2019; Sendinc et al., 2019). One study used the CRISPR-Cas9 system to target exons of FTO to generate a 293T FTO^−/−^ cell line and compared the differential expression profiles of FTO-bound genes with those of all genes and found that there were no significant differences between gene expression profiles and that regulation of gene expression and mRNA stability may not be a major role for nuclear FTO (Bartosovic et al., 2017). ALKBH5 is the second demethylase identified to date that uses m^6^A as the only known substrate and is comparable to the m^6^A demethylation activity of FTO (Zheng et al., 2013; Yang et al., 2018).

### 2.3 m^6^A Reader

The m^6^A reading proteins include proteins containing the YTH structural domain and the family of intranuclear inhomogeneous nuclear ribonucleoproteins (HNRNP), etc., which can directly bind to the motif sequence of m^6^A and participate in the processes of mRNA precursor shearing, mRNA degradation, translation, and translocation, and non-coding RNA biogenesis.

#### 2.3.1 YTH Family Proteins

The mammalian genome contains five YTH structural domain-containing proteins, including YTHDF family proteins 1–3 (YTHDF1-3); YTHDC family proteins 1-2 (YTHDC1-2). YTHDF family members are mainly located in the cytoplasm, and the three YTHDFs have different functions, with YTHDF2 being the first to be identified “reader” that accelerates mRNA degradation by binding to m^6^A at the mRNA 3′UTR and localizing it to processing vesicles (P-bodies) to recruit RNA degradation enzymes (Lee et al., 2020). A recent study showed that under stress conditions, complexes containing m^6^A and YTHDF proteins assign to different endogenous phase-separated compartments, such as P-bodies, stress granules, or neuronal RNA particles, and that m^6^A-mRNA stability and translation are regulated by liquid-liquid phase separation (Ries et al., 2019). YTHDF1 binds to the m^6^A-site around the stop codon and enhances mRNA translation by recruiting the eIF3 translation initiation complex rather than by m^7^G cap-dependent means (Wang et al., 2015). YTHDF3 can interact with YTHDF1 to improve RNA translation efficiency and bind to YTHDF2 to promote RNA degradation (Shi et al., 2017; Zaccara et al., 2019; Wiener and Schwartz, 2021). Notably, depletion of YTHDF3 reduces the binding of YTHDF1 and YTHDF2 to target transcripts, and loss of YTHDF1 or YTHDF2 similarly reduces the amount of RNA bound by YTHDF3, suggesting an important role for YTHDF3 in RNA-specific binding YTHDF1/2 (Zhao et al., 2020). In contrast to the prevailing view that “different m^6^A sites bind different YTHDF proteins,” some researchers found that the YTHDF paralogs are highly similar in sequence, functional domain, interacting proteins, and intracellular localization. All m^6^A sites bind to all three YTHDF proteins similarly, and they act redundantly to induce degradation of the same subset of mRNAs, with no evidence that they directly promote translation (Zaccara and Jaffrey, 2020). YTHDC1 is predominantly located in the nucleus and directly recruits splicing factor serine and arginine-rich splicing factor 3 (SRSF3) in the nucleus while blocking the binding of SRSF10 to regulate selective splicing of precursor RNAs (Xiao et al., 2016; Patil et al., 2018). YTHDC2, the largest member of the YTH family, also binds preferentially to m^6^A within the shared motif to enhance translation efficiency (Mao et al., 2019).

#### 2.3.2 HNRNP Family Proteins

**FIGURE 1:**
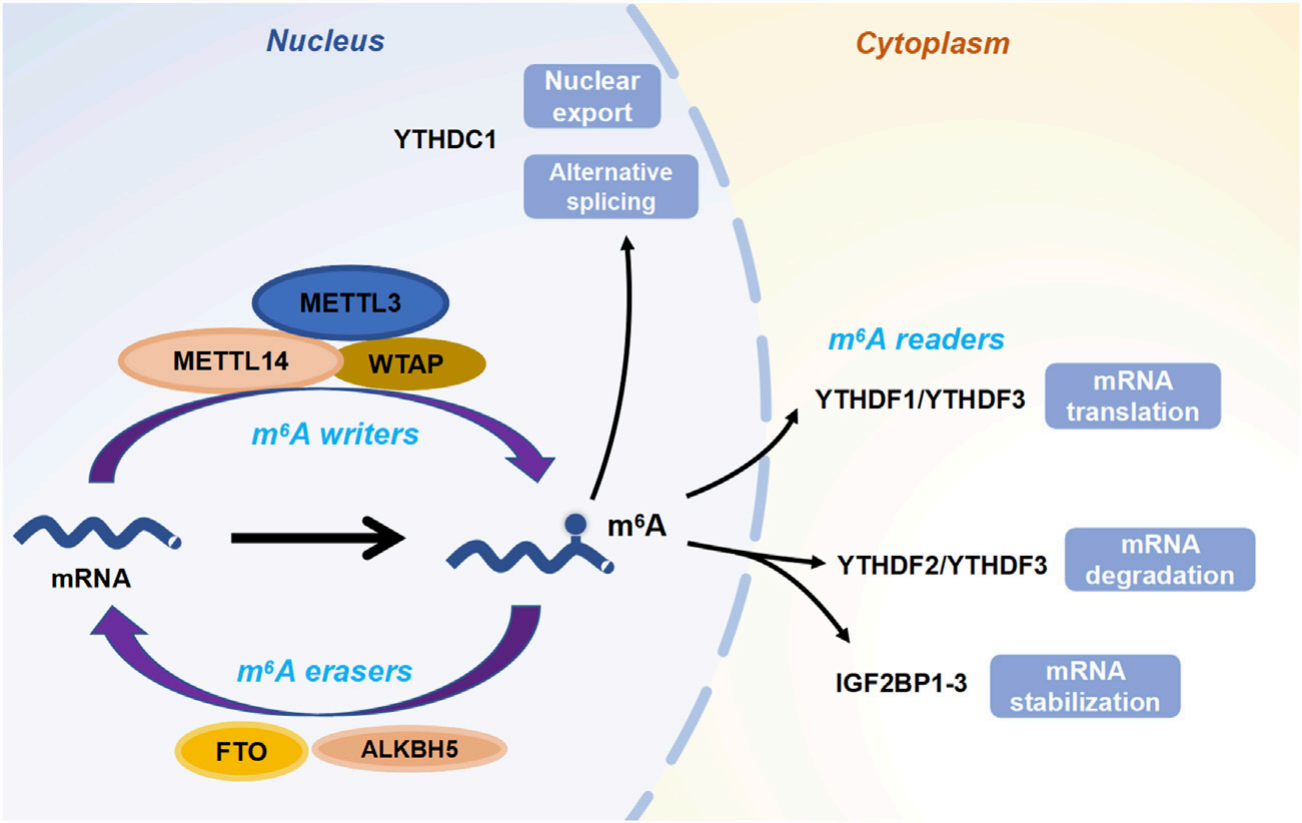
The dynamic and reversible processes of m^6^A modification. “Writers” deposit m^6^A methylation on RNAs, while “erasers” remove the m^6^A marks. Then “readers” are responsible for regulating the fate of targeted RNAs.

In the nucleus, HNRNPC functions as an m^6^A reader by binding to the unstructured m^6^A switch region and regulating splicing (Zarnack et al., 2013; Liu et al., 2015). HNRNPA2B1 is similar to YTHDC1 in mediating selective splicing (Alarcón et al., 2015; Zhao et al., 2017; Zhou et al., 2019). In addition to m^6^A readers containing the YTH structural domain, several other RNA-binding proteins have been reported to bind m6A-containing RNAs preferentially. IGF2BPs are unique and conserved m^6^A readers, and IGF2BP1-3 enhances the stability and improves the translation efficiency of m^6^A-modified mRNAs and enhances mRNA stabilization by recruiting their cofactors HuR and MATR3 effects (Huang et al., 2018) (Figure 1).

## 3 Histones and Histone Modifications

The basic unit of chromatin is the nucleosome core particle, which contains DNA and histone octamers. The histone octamer consists of a central heterotetramer of histones H3 and H4 flanked by two heterodimers of histones H2A and H2B. Histones are subject to numerous post-translational modifications, including acetylation and methylation of lysine (K) and arginine (R), phosphorylation of serine (S), and threonine (T), and ubiquitination of lysine. Each lysine residue can receive one, two, or even three methyl groups, while arginine can be monomethylated or dimethylated (Peterson and Laniel, 2004).

Histone modifications can control chromatin fibers’ structure and function, and different modifications can produce different results. Site-specific combinations of histone modifications are closely related to specific biological functions. Combinations of H4K8 acetylation, H3K14 acetylation (Shvedunova and Akhtar, 2022), and H3S10 phosphorylation (Komar and Juszczynski, 2020) are usually associated with transcription. In contrast, trimethylation of H3K9 (Ninova et al., 2019) and the lack of H3 and H4 acetylation (Shvedunova and Akhtar, 2022) are associated with transcriptional repression in higher eukaryotes. Specific patterns of histone modifications are also associated with global chromatin dynamics, with diacetylation of histone H4 at K4 and K12 associated with histone deposition in the S phase, and phosphorylation of histones H2A (at S1 and T119) and H3 (at T3, S10, and S28) involved in transcriptional regulation and chromatin densification (Kouzarides, 2007).

## 4 Crosstalk Between m^6^A Modifications and Histone Modifications

The level of m^6^A modification depends on the intrinsic preference of METTL3/METTL14 methyltransferase for specific nucleotide sequences and the extrinsic regulation of methyltransferase complex activity by external factors such as RNA-binding proteins, transcription factors, and RNA polymerases. When external determinants dominate, m^6^A modification levels may respond to changes in external factors in different cellular states. It implies that m^6^A modifications are intertwined with other cellular processes that allow loci to gain or lose methylation in response to specific cellular events (He and He, 2021).

The interaction between histone modifications and RNA methylation represents a new level of regulation and complexity in the regulation of gene expression. Central to the precise and synchronous regulation of gene expression is the complex crosstalk between multiple steps involved in transcript biosynthesis and processing. m^6^A serves as a multifunctional checkpoint that can couple different levels of gene regulation to each other. And the crosstalk between histone modifications and m^6^A modifications also becomes an important external factor in the regulation of m^6^A modifications.

### 4.1 Histone H3K36me3 Modification Directs the Deposition of m^6^A in CDS and 3′ UTR

Histone H3 lysine 36 trimethylation (H3K36me3) is a transcriptional elongation marker, mainly distributed in the coding sequence (CDS) and 3′ UTR. H3K36me3 has a similar CDS and 3′UTR distribution pattern to m^6^A. Further analysis of the ChIP-seq data of the m^6^A epigenome and H3K36me3 in the same cells confirmed that 69.2% of m^6^A peaks overlapped with the H3K36me3 modification. More importantly, knockdown of H3K36me3 methyltransferase SETD2 or overexpression of demethylase KDM4A decreased cellular H3K36me3 levels and significantly reduced m^6^A modification levels in total RNA and poly (A) RNA in human and mouse transcriptomes, suggesting that H3K36me3 can regulate m6A deposition. Notably, more than 80% of H3K36me3-dependent m^6^A sites are also targeted sites of METTL3, METTL14, and WTAP, suggesting that H3K36me3-mediated regulation of m^6^A deposition has a broad impact on the transcriptome (Huang et al., 2019). Mechanistically, m^6^A MTC interacts with H3K36me3 and RNA Pol II. H3K36me3 is directly recognized and bound by METTL14, when encountering RNA Pol II, recruits other components of m^6^A MTC and mediates the deposition of m^6^A on newly synthesized RNA. Thus, recognizing H3K36me3 by METTL14 and specific recognition of DRACH motifs by m^6^A MTC enables precise and dynamic deposition of m^6^A on the transcriptome and reveals the importance of METTL14 in the selective and precise deposition of m^6^A (Huang et al., 2020).

### 4.2 H3K4me3 Modification and m^6^A Methyltransferase

The activity of m6A methylesterase can affect the expression levels of histone H3K4 methylesterases such as SETD1A, SETD1B, and KMT2D. H3K4me3, as a promoter mark, is mainly enriched in the promoter region near the transcription start site and is associated with gene activation. Knockdown of METTL14, METTL3, and WTAP in erythroid cells resulted in a substantial loss of H3K4me3 signaling at the transcription start site and was particularly pronounced after knockdown METTL14 and WTAP (Kuppers et al., 2019). In addition, METTL14 expression was found to be decreased in colorectal cancer tissues and negatively correlated with the expression level of histone demethylase KDM5C, which catalyzes H3K4me2/3 demethylation and thus represses gene transcription. Knockdown of KDM5C significantly increased the expression level of METTL14. ChIP results showed that the promoter of METTL14 is enriched in H3K4me3. Mechanistically, KDM5C mediates the demethylation of H3K4me3 in the promoter region of METTL14 and represses METTL14 transcription. After the knockdown of KDM5C, H3K4me3 modification in the METTL14 promoter increased and activated METTL14 expression (Chen et al., 2020).

### 4.3 m^6^A Co-transcription Directs H3K9me2 Demethylation and Promotes Gene Expression

There is a strong correlation between m^6^A RNA methylation sites and H3K9me2 distribution in mouse embryonic stem cells. To clarify the direct effect of m^6^A on histone modifications, mutating the core components of m^6^A methyltransferase METTL3/METTL14 and demethylase FTO, deletion of METTL3 or METTL14 did not significantly alter the expression of H3K9me2 methyltransferases G9a and GLP and demethylase KDM3A/B/C but led to an increase in H3K9me2 levels increased; in contrast, deletion of FTO led to a decrease in H3K9me2 levels. It was further found that KDM3B protein expression correlated most strongly with METTL3/METTL14, and 70.5% of KDM3B-modified genes overlapped with m^6^A peaks. Although the loss of METTL3 catalytic activity did not affect KDM3B expression, it resulted in reduced binding of KDM3B to chromatin, especially in the chromatin region where m^6^A was deposited. Most YTHDC1 co-localizes with KDM3B in the nucleus. Mechanistically, the m^6^A recognition protein YTHDC1 and the H3K9me2 demethylase KDM3B interact, and YTHDC1 can recruit KDM3B to m6A-related regions, thereby promoting H3K9me2 demethylation and gene expression at the corresponding sites, and ultimately H3K9me2-regulated genes (Li et al., 2020).

METTL3 promotes the translation of mRNA through different mechanisms: METTL3 interacts with eIF3h to promote the cyclization of mRNA, resulting in increased ribosomal efficiency and thus enhanced translation efficiency of target mRNA, independent of its catalytic activity (Choe et al., 2018). YTHDF1 binds to m^6^A-containing mRNAs and drives translation in an m^6^A-dependent manner. On the other hand, YTHDF3 can also enhance translation efficiency by binding to YTHDF1 and eIF4A3, and YTHDF3 and YTHDF1 cooperate during mRNA translation (Zaccara et al., 2019; Wiener and Schwartz, 2021). In addition, IGF2BP1/2/3 can also accelerate mRNA translation (Huang et al., 2018).

### 4.4 Histone Demethylase KDM4C Promotes ALKBH5 Expression and Reduces H3K9me3 Levels

KDM4C expression levels in leukemic cells from acute myeloid leukemia patients are positively correlated with ALKBH5 expression levels. Histone demethylase KDM4C is enriched in the ALKBH5 promoter region, regulates ALKBH5 expression by increasing chromatin accessibility of ALKBH5, and promotes transcription factors MYB and Pol II by decreasing H3K9me3 levels recruitment. Knockdown of KDM4C significantly downregulated mRNA and protein levels of ALKBH5 in leukemic cells while leading to the accumulation of the repressive histone modification H3K9me3, accompanied by a decrease in MYB and Pol II-CTD binding. In conclusion, KDM4C regulates ALKBH5 expression in leukemic cells by increasing chromatin accessibility and promoting the binding of MYB and Pol II-CTD to the ALKBH5 promoter (Wang et al., 2020).

### 4.5 m^6^A Methyltransferase Affects H3K27me3 Modification by Regulating EZH2 Expression and Also Affects H3K27ac Modification by Regulating CBP and p300

METTL3 regulates the decay of histone methyltransferase EZH2 mRNA in an m^6^A-dependent manner, the METTL3 motif binds to EZH2 and is enriched for H3K27ac but not H3K27me3, and knockdown of EZH2 reduces the H3K27ac modification of the METTL3 promoter, suggesting that EZH2 binds to the METTL3 promoter and acts in an H3K27ac dependent manner as an activator of METTL3 expression (Li et al., 2021). It has also been shown that the presence of m^6^A on EZH2 transcripts increases H3K27me3 levels and that knockdown of METTL3 decreases EZH2 protein expression and H3K27me3 levels (Chen et al., 2019). Other researchers knocking down METTL14 in neural stem cells found increased levels of H3K27ac, H3K4me3, and H3K27me3 modifications, and mechanistically, METTL14-mediated methylation of m^6^A reduced the stability of histone acetyltransferase CREB-binding protein and p300 transcripts, thereby regulating histone H3K27ac modifications (Wang et al., 2018). Crosstalk exists between m^6^A and H3K27me3 during bacterial infection, and mRNA for the histone demethylase KDM6B is modified by m^6^A and degraded by YTHDF2. YTHDF2 deficiency stabilizes KDM6B to promote H3K27me3 demethylation of multiple pro-inflammatory cytokines and subsequently enhances transcription of pro-inflammatory factors; in summary, KDM6B recruits the m^6^A methyltransferase complex to promote m^6^A methylation in mRNA by removing the adjacent H3K27me3 barrier (Wu et al., 2020).

Blocking METTL3 or METTLE14 reverses m^6^A modification, reduces mRNA degradation efficiency, and enhances gene expression (Mauer et al., 2017). The C-terminal YTH domain of YTHDF2 protein binds to mRNA containing m^6^A while the N-terminal domain locates the mRNA as an “RNA degradation agent” for further degradation (Wang et al., 2014; Wang et al., 2015). YTHDF2 mediates mRNA degradation mainly through two different pathways: YTHDF recruits HRSP12, which in turn mediates the cleavage of target molecules by the RNA endonuclease RNase P/MRP complex (Park et al., 2019; Lee et al., 2020), YTHDF2 also recruits the CCR4-NOT deadenylase complex by directly interacting with the SH structural domain of CNOT1 to initiate mRNA decay (Du et al., 2016). The IGF2BP-mediated promotion of mRNA stability by m^6^A “reading” was recently reported, and IGF2BP may bind to mRNA-stabilizing proteins (e.g., HuR, MATR3, and PABPC1) to enhance mRNA stability (Huang et al., 2018).

## 5 Conclusion and Perspectives

There has been an explosion of research on m^6^A, with increasing evidence confirming the importance of m^6^A in regulating gene expression and disease progression. m^6^A modifications can couple different levels of gene regulation to each other, and the focus of research on m^6^A modifications has now gradually shifted to studying the interactions between RNA methylation and other epigenetic regulatory players. It is essential to understand the defects in such interactions or crosstalk and how they lead to the development of various diseases is of great interest. Despite the increasing number and depth of studies, we need to continue to delve deeper into the molecular basis of m^6^A effects.

The dilemma of developing the m^6^A sequencing method for RNA: firstly, RNA must be reverse transcribed to cDNA for amplification and sequencing, and the site information of m^6^A will be lost during reverse transcription; secondly, although m^6^A is the most abundant modification on eukaryotic mRNA, the absolute content is still shallow, and non-coding RNAs such as rRNA and tRNA also contain m^6^A modification. It is crucial to distinguish the highly similar m^6^A and m^6^Am modifications. miCLIP, PA-m^6^A-seq, m^6^A-CLIP, and other UV cross-linked immunoprecipitation techniques have been improved to address the shortcomings that meRIP-seq can only isolate m^6^A-rich regions and cannot accurately distinguish m^6^A modifications, can more accurately distinguish m^6^A RNA methylation at single-nucleotide resolution, and provide higher resolution transcriptome-wide profiles of m^6^A RNA methylation (Linder et al., 2015). However, currently available methods for m^6^A analysis typically require large amounts of RNA. The development of new techniques that require only a limited amount of RNA material and provide base-resolved m^6^A profiles with better quantitative information would greatly advance research in research this area.
